# Detection of major anomalies during the first and early second trimester: Single-center results of six years

**DOI:** 10.4274/jtgga.2017.0125

**Published:** 2018-08-06

**Authors:** Erol Arslan, Selim Büyükkurt, Mete Sucu, Mehmet Özsürmeli, Selahattin Mısırlıoğlu, S. Cansun Demir, İ. Cüneyt Evrüke

**Affiliations:** 1 Department of Obstetrics and Gynecology, Unit of Perinatology, Çukurova University School of Medicine, Adana, Turkey

**Keywords:** First trimester, anomaly, ultrasound, termination

## Abstract

**Objective:**

Fetal structural malformations affect approximately 2-3% of all pregnancies. Only focusing on trisomy screening in first trimester and deferring the anatomic screening to second trimester may result with late detection of major anomalies that can be diagnosed earlier with careful examination.

**Material and Methods:**

This was a descriptive study of retrospective data that were obtained from all terminated single pregnancies due to ultrasonographic findings of major anomalies from 2011 to 2016 in our department. The study was based on a chart review and only abnormalities that were diagnosed before the 16^th^ week were included.

**Results:**

Two hundred forty-four first trimester pregnancy terminations were performed. In total, 273 anomalies were detected in the 244 patients. Cranial NTD comprised 32% of all anomalies (n=89). Fifteen percent of anomalies (n=41) needed detailed anatomic scanning for early diagnosis.

**Conclusion:**

In this study, we presented the number and percentage of our early diagnosed anomalies by years, as well showed our diagnostic performance for specific anomalies such as atrioventricular septal defect during a 5-year period. The study provides valuable information for future studies in Turkey and shows the need for an anatomic scan protocol while performing aneuploidy screening during early gestation.

## Introduction

Major congenital anomalies affect 2-3% of all pregnancies (1). Although second trimester ultrasound screening between the 18-23^th^ gestational weeks has routinely been used for anomaly screening (2), it is widely accepted that most anomalies can be detected earlier. In this aspect, major abnormalities have been separated into three groups due to the probability of their detection rate by 11-14^th^ weeks’ ultrasound (3). The first group includes anomalies that can be easily detected in the first trimester such as anencephaly, the second group comprises anomalies that reveal ultrasonography signs later in gestation and have no possibility of early detection such as hypoplasia of cerebellum. The third group anomalies can be detected in first trimester with meticulous examination using high-tech devices. This group of anomalies includes spina bifida occulta, skeletal dysplasia, and some kinds of cardiac defects, which sometimes need to be examined using transvaginal ultrasound (3).

First trimester ultrasound has mostly been used for confirmation of fetal viability, establishment of gestational age, measurement of nuchal translucency (NT), and for first trimester screening (4). Improvements in sonographic technology and ultrasonography scanners with high-resolution imaging, as well increased experience of sonographers regarding fetal imaging, have enabled the concept of early screening of fetal structural malformations in the late first trimester (5,6). Furthermore, early fetal echocardiogram has been defined for echocardiography implemented before the 16^th^ gestational week for investigating congenital heart defects, especially in patients with high risk (7). 

Screening in the first and early second trimester and early detection of major anomalies will lead to early decisions of pregnancy termination. Moreover, doing it before the 16^th^ gestational week brings the advantage of early termination before women feel movements of the fetus. This is important for women, especially in cultures in which beliefs strongly affect social life rules. Furthermore, early termination of pregnancy has physical and physiological advantages for women and their families compared with late termination. 

In this study, we aimed to investigate the major anomalies that resulted with termination of pregnancy before the 16^th^ gestational week by years, and so to find their distribution during a six-year period. Moreover, we tried to ascertain whether early anatomic screening during the first and early second trimester might result in determining anomalies such as atrioventricular septal defect (AVSD) and spina bifida, which were ranked in the third group of anomalies by Syngelaki et al. (3), as discussed previously.

## Material and Methods

This was a retrospective study of all terminated single pregnancies due to ultrasonographic findings of major congenital anomalies from 2011 to 2016 that were diagnosed in our department. This was a descriptive study and based on chart review and only anomalies that were diagnosed at or before 16^+0^ gestational weeks were included. Only major anomalies that required termination of pregnancy were included and diagnoses were confirmed by fetal autopsy. Ultrasonographic findings such as thickened NT, nasal bone hypoplasia, tricuspid regurgitation, and reversed a-wave on ductus venosus Doppler were excluded. Women who did not accept termination or who underwent termination outside of our center were excluded. The patients’ ages and gestational weeks are presented as mean and standard deviation. This study was approved by the local ethics committee of our university.

## Results

The women’s mean age was 28.8±6.1 years and the mean gestational age at termination was 13^+5/7^±1^+3/7 ^weeks. A total of 244 pregnancies were terminated at ≤16^+0^ weeks from 2011 to 2016. Two hundred seventy-three anomalies were detected among the 244 patients during the study period. Although 218 patients had isolated anomalies, 26 of 244 patients had at least two different anomalies. The detected anomalies and their distribution by years are shown in Table 1. Cranial neural tube defects (NTD) such as anencephaly and encephalocele accounted for 32.6% of all detected anomalies (n=89). These were followed by cystic hygroma (23%, n=63). Two hundred thirty-two anomalies could easily be diagnosed during early gestation; however, 41 of 273 (15%) anomalies (14 skeletal dysplasia, 9 spina bifida occulta, 7 multicystic renal dysplasia, 6 AVSD, 2 hypoplastic left heart, 1 ductus venosus agenesia, 1 sacroccocygeal teratoma, 1 micrognathia) belonged to third group of anomalies (Table 1).

## Discussion

In the present study, it was observed that the number of diagnosed anomalies increased by years except in 2014, which had a lower number of abnormalities compared with 2013. The higher numbers of abnormalities were related with high numbers of cranial NTD detected in 2013, which were 2-3 times higher than in other years. Excluding this peak in 2013, there was a linear increase from 2011 to 2016. This might be simply explained by an arbitrary increase in the number of anomalies; nevertheless, more detailed anatomy screening could be the cause of this increase from 2011 to 2016.

Cranial NTD comprised 31% of major structural anomalies that were terminated from 2011 to 2016 and it was the most common abnormality during the study period, followed by cystic hygroma, which is consistent with the literature (8). Forty-one of the 273 (15%) anomalies that were diagnosed before the 16^th^ gestational week were classified in third group of anomalies as described by Syngelaki et al. (3). These sorts of anomalies needed a detailed examination, as well transvaginal ultrasound evaluation in some cases for early detection. Although 15% was not a small rate among the total anomalies considering the extremely high percentage of cranial NTD and cystic hygroma, we still believe that more anomalies could be detected by using an anatomic screening protocol during the first trimester screening, in addition to using transvaginal ultrasound at least in suspected cases.

In our study, we determined the 16^th^ gestational week as cutoff beside the 14^th^ gestational week for various reasons. First, our primary aim was not to recognize all abnormalities during the first trimester aneuploidy screening, but to diagnose major anomalies that would undergo termination of pregnancy as early as possible, preferably before the mother could feel fetal movements, which might make the decision for termination easier. Second, examining the fetal heart and other body parts in the first trimester was not appropriate for most of the patients, when it was considered that examination by transvaginal ultrasound was not a favorable method for pregnant women in our country. Moreover, studies in the literature used both the 14^th^ and 16^th^ gestational weeks as cutoff points for early anomaly screening (7,9). Four of 6 AVSD cases and all hypoplastic left heart syndrome cases (n=2) were diagnosed before the 14^th^ gestational week, which might encourage us to perform more early scanning of cardiac defects, at least for gross pathologies that can give signs in earlier gestational weeks. Moreover, there were 9 cases of spina bifida and 14 cases of lethal skeletal dysplasia in total that were diagnosed before the 16^th^ gestational week. As discussed previously, these anomalies were grouped in the literature as anomalies that could be diagnosed by meticulous screening, and our results were encouraging regarding early diagnosis of these anomalies.

Several studies in the literature, those searching for the effectiveness of early anomaly screening, were designed prospectively and compared the first trimester screening with 18-24^th^ week screening (10,11). In addition, some other studies retrospectively looked over the early anomaly screening, as in our study (8). Those studies determined that early anomaly screening could diagnose nearly 50% of major abnormalities in unselected patients, and 61% of all kinds of abnormalities in high-risk patients (8). In our study, we could not categorize patients according to their risk factors because we did not have enough information. Likewise, our data could not reveal a percentage of anomalies that were diagnosed during the first trimester in a selected population because of its retrospective design and the fact that it only included terminated pregnancies in our center. It should be born in mind that some patients did not accept termination or underwent termination in other centers. Different than those studies, we investigated specific anomaly rates among total anomalies.

To the best of our knowledge, this is the first study to investigated early diagnosis of fetal anomalies in Turkey. Despite its retrospective characteristic and indirect measurement of early anomaly screening effectiveness, it provides valuable information for future studies in our country. Furthermore, this study shows the need for an anatomic scan protocol while performing aneuploidy screening during early gestation in our country.

## Figures and Tables

**Table 1:**
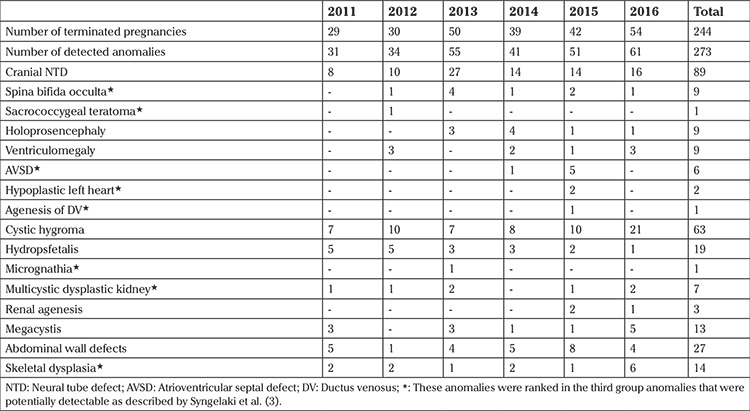
The number of patients and diagnosed anomalies and their distributions from 2011-2016
